# Supplementary material for: Alpha protocadherins and Pyk2 kinase regulate cortical neuron migration and cytoskeletal dynamics via Rac1 GTPase and WAVE complex in mice
Source: eLife. 2018 Jun 18;7:e35242. doi: 10.7554/eLife.35242 (PMC6047886; doi:10.7554/eLife.35242)
Supplement: Supplementary file 1. [file elife-35242-supp1.docx]

**Supplementary File 1. Oligos Used and Their Sequences**

| **Oligos for plasmid construction** | |
| --- | --- |
| SCR_F | CCGGCAACAAGATGAAGAGCACCAACTCGAGTTGGTGCTCTTCATCTTGTTGTTTTTG |
| SCR_R | AATTCAAAAACAACAAGATGAAGAGCACCAACTCGAGTTGGTGCTCTTCATCTTGTTG |
| αKD1_F | CCGGAACAGTATCCAGTGCAACACCCTCGAGGGTGTTGCACTGGATACTGTTTTTTTG |
| αKD1_R | AATTCAAAAAAACAGTATCCAGTGCAACACCCTCGAGGGTGTTGCACTGGATACTGTT |
| αKD2_F | CCGGAATTCATTATCCCAGGATCTCCTCGAGGAGATCCTGGGATAATGAATTTTTTTG |
| αKD2_R | AATTCAAAAAAATTCATTATCCCAGGATCTCCTCGAGGAGATCCTGGGATAATGAATT |
| Pcdhα6_EcoRV_F | CGGATATCGCCACCATGGATTTTACCACTGAAG |
| Pcdhαc1_EcoRI_F | GGAATTCGCCACCATGGTGGGCTGGGGAATGG |
| Pcdhα6_αc1_KpnI_R | ATGGTACCCTGGTCACTGTTGTCCGTCG |
| Pcdhαc2_EcoRI_F | GGAATTCGCCACCATGGAGCAGGCGGGAGCCAG |
| Pcdhαc2_BamHI_R | CGGGATCCAGCTGGTCACTGTTGTCCGTC |
| αKD2_resistant_F | CAGACAAGTTTATCATTCCTGGTTCTCCTTGCAATC |
| αKD2_resistant_R | CAGGAGAACCAGGAATGATAAACTTGTCTGGCAAC |
| Myr_EcoRI_F | CGGAATTCGCCACCATGGGCAGTAGCAAG |
| PcdhαCD_BglII_R | GATGAGTTTTTGTTCAGATCTCTGGTCACTGTTGTCCGT |
| Myr_α6_ICD_F | GCCGGCGTCGGTGCTCAGTTCCACCTAC |
| Myr_α6_ICD_R | CTGAGCACCGACGCCGGCGCTGGCTG |
| Myr_αc1_ICD_F | GCGCCGGCGTAAGTTGAACCAGAGCCCA |
| Myr_αc1_ICD_R | GGTTCAACTTACGCCGGCGCTGGCTGGG |
| Myr_αc2_ICD_F | GCCGGCGTAAGTGCTACCGCTACACTGC |
| Myr_αc2_ICD_R | GGTAGCACTTACGCCGGCGCTGGCTG |
| WAVE2_HindIII_F | CCAAGCTTGCCACCATGCCGTTAGTAACCAGGAAC |
| WAVE2_BglII_R | GAAGATCTATCCGACCAGTCGTCTTCATC |
| Abi2_EcoRI_F | GGAATTCGCCACCATGGCGGAGCTGCAGATG |
| Abi2_BglII_R | GAAGATCTCTCCGAATAATGCATGATGG |
| Pcdhα_WIRS_AA_F | TTTATAGCCGCTGGCAAAAAGGAG |
| Pyk2_KD_F | CCGGCAAGGAGAAGTTCATGAGTGACTCGAGTCACTCATGAACTTCTCCTTGTTTTTG |
| Pyk2_KD_R | AATTCAAAAACAAGGAGAAGTTCATGAGTGACTCGAGTCACTCATGAACTTCTCCTTG |
| Pyk2_EcoRI_F | CGGAATTCGCCACCATGTCCGGGGTGTCTGA |
| Pyk2_BglII_R | GAAGATCTTCACAGATCCTCTTCTGAG |
| Rac1_EcoRI_F | CGGAATTCGCCACCATGCAGGCCATCAAGTGTG |
| Rac1_BglII_R | GAAGATCTTTACAACAGCAGGCATTTTCTC |
| Rac1_Q61L_R | AATCTTCCAGTCCAGCTGTGTCCCATAG |
| Rac1_G12V_R | GGTGGTGGGAGACGTAGCTGTTGGTAAAACC |
| NeuroD_Pyk2_XhoI_F | CCGCTCGAGCCACCATGTCCGGGGTGTCT |
| NeuroD_Pyk2_XmaI_R | GGGCCCGGGTCACAGATCCTCTTCTGAGATGAG |
| Pyk2_ΔFERM_EcoRI_F | TGGAATTCCCACCATGAAGAAAGATGGTGAGAAG |
| Pyk2_ΔFAT_BglII_R | GAAGATCTGTTGGCTGTGGGCTGAATG |
| FERM_BglII_R | GAAGATCTGGCATGCATGATGAGAGAGC |
| **Primers for gene expression analyses** | |
| M_Pcdhα1_F | GAACATAGCGGAAAGAAGTGAC |
| M_Pcdhα2_F | AATCAGCAGAAGAGAGACAACC |
| M_Pcdhα3_F | CTTACACCATGCCCAGTTAATC |
| M_Pcdhα4_F | TCAAGGGACAGAGAGGATCAA |
| M_Pcdhα5_F | GAACCTCTGGTTCAGACTCCAC |
| M_Pcdhα6_F | GGGTGAGCATCAGGATTTG |
| M_Pcdhα7_F | AGCCTCCAGAGTGGATCAGA |
| M_Pcdhα8_F | CAGAACCATCTGTTTCTTTGGA |
| M_Pcdhα9_F | GAAGTGGGAATGGAAAGTCATT |
| M_Pcdhα10_F | TGGTTTGGGTTCTGGAGATAGT |
| M_Pcdhα11_F | GGGTAGAGATGAAAGGGAAAGAC |
| M_Pcdhα12_F | TGTGTTAGGCTCTGCAGAGGAC |
| M_Pcdhαc1_F | TTCGGAATAGGAAAGGGGATCA |
| M_Pcdhαc2_F | GCACAGTACCGGGAACCTGATT |
| M_PcdhαCon_F | GAGGAGGCTGGCATTCTACG |
| M_PcdhαCon_R | CGGATGGAGATGATTGCAGGAG |
| **Oligos for generation of CRISPR mice** | |
| Pyk2KO_sgRNA_F | TAATACGACTCACTATAGGGGGCACTTTACGCCGGCCTGAGTTTTAGAGCTAGAAATAG |
| Pyk2Y402F_sgRNA_F | TAATACGACTCACTATAGGGtacagAGTCAGACATCTATGGTTTTAGAGCTAGAAATAG |
| sgRNA_R | GCACCGACTCGGTGCCACTT |
| Pyk2Y402F_ssODN | gggctgggcccctttctgtcattacagAGTCAGACATCTTTGCAGAGATTCCCGATGAGACCCTGCGAAGACCAGGAGg |
| **Oligos for genotyping of Pyk2 CRISPR mice** | |
| Pyk2KO_F | GGATGTGGCATGTGGCTTGCAAGAG |
| Pyk2KO_R | TACCTGGATCTCTGTCTGCACTGTG |
| Pyk2Y402F_F | ACTGTGTGGCTTCCTTGAATCCTGG |
| Pyk2Y402F_R | TCTCCTGTGGTGTCCCATGAATAC |
